# Identification of second primary tumors from lung metastases in patients with esophageal squamous cell carcinoma using whole-exome sequencing

**DOI:** 10.7150/thno.45311

**Published:** 2020-08-25

**Authors:** Liyan Xue, Weihua Li, Xinyi Fan, Zitong Zhao, Wei Zhou, Zhimin Feng, Linxiu Liu, Hua Lin, Lin Li, Xuemin Xue, Xuanlin Huang, Peide Huang, Jia Guo, Peina Du, Ning Lu, Lin Li, Qimin Zhan, Yongmei Song

**Affiliations:** 1 Department of Pathology, National Cancer Center/National Clinical Research Center for Cancer/Cancer Hospital, Chinese Academy of Medical Sciences and Peking Union Medical College, Beijing, 100021, China; 2 State Key Laboratory of Molecular Oncology, National Cancer Center/National Clinical Research Center for Cancer/Cancer Hospital, Chinese Academy of Medical Sciences and Peking Union Medical College, Beijing, 100021, China; 3 Department of Medical Record, National Cancer Center/National Clinical Research Center for Cancer/Cancer Hospital, Chinese Academy of Medical Sciences and Peking Union Medical College, Beijing, 100021, China; 4 Center for Cancer Precision Medicine, Cancer Hospital, Chinese Academy of Medical Sciences and Peking Union Medical College, Beijing, 100021, China; 5 Laboratory of Molecular Oncology, Peking University Cancer Hospital, Beijing 100142, China; 6 Shanghai Clinical Center for Endocrine and Metabolic Diseases, Shanghai Key Laboratory for Endocrine Tumors, Rui-Jin Hospital, Shanghai Jiao-Tong University School of Medicine, Shanghai, 200025, China

**Keywords:** esophageal squamous cell carcinoma, lung metastasis, second primary tumor, whole-exome sequencing, clonal relationship

## Abstract

Esophageal squamous cell carcinoma (ESCC) patients with a synchronous or metachronous lung tumor can be diagnosed with lung metastasis (LM) or a second primary tumor (SPT), but the accurate discrimination between LM and SPT remains a clinical dilemma. This study aimed to investigate the feasibility of using the whole-exome sequencing (WES) technique to distinguish SPT from LM.

**Methods:** We performed WES on 40 tumors from 14 patients, including 12 patients with double squamous cell carcinomas (SCCs) of the esophagus and lung (lymph node metastases were sequenced as internal controls) diagnosed as LM according to pathological information and 2 patients with paired primary ESCC and non-lung metastases examined as external controls.

**Results:** Shared genomic profiles between esophageal (T) and lung (D) tumors were observed in 7 patients, suggesting their clonal relatedness, thus indicating that the lung tumors of these patients should be LM. However, distinct genomic profiles between T and D tumors were observed in the other 5 patients, suggesting the possibility of SPTs that were likely formed through independent multifocal oncogenesis.

**Conclusions:** Our data demonstrate the limitations and insufficiency of clinicopathological criteria and that WES could be useful in understanding the clonal relationships of multiple SCCs.

## Introduction

Esophageal cancer is one of the most common malignancies worldwide 1. It can be further divided into two main histologic subtypes: esophageal adenocarcinoma and esophageal squamous cell carcinoma (ESCC). The incidence of ESCC exhibits significant geographical differences, and approximately half of newly diagnosed ESCCs each year occur in China 2. Regional lymph node (LN) or distant metastases of ESCC are usually present at the time of diagnosis, and a high frequency of metastases account for the poor prognosis of ESCC 3. The lung is one of the most common metastatic sites 4, 5. However, lung squamous cell carcinoma (LUSC) in a patient with ESCC could be either metastasis or a second primary tumor (SPT). Lung metastasis (LM) is formed through hematogenous metastasis as a late stage of ESCC, while SPT is generally believed to originate from independent clones, suggesting an early stage of cancer. Thus, further chemotherapy is needed for LM, which might be considered overtreatment in SPT cases 6. Therefore, it is of great importance to distinguish SPT from LM since the diagnosis can affect subsequent treatment strategies and survival evaluations.

Several clinicopathological criteria, such as the disease-free interval, the numbers and locations of lung lesions, adjacent precursor lesions, and histological similarity, have been adopted to discriminate between primary and metastatic tumors in patients with multiple tumors at presentation 7, 8. Nevertheless, these criteria are usually insufficient or not reliable for accurate diagnostics, and more importantly, such a dilemma makes it difficult to decide the subsequent management for patients after the curative resection of multiple tumors.

Based on the clonal evolution theory, two tumors derived from a common clone suggest metastasis, while distinct clones indicate multiple primary cancers. Thus, several molecular markers, such as *TP53* mutations 9, 10, P53/P16 immunohistochemistry 11, HPV genotyping 12, 13, gene expression 14, and loss of heterozygosity (LOH) involving microsatellite markers 15, have been analyzed to distinguish SPT from LM in patients with double squamous cell carcinomas (SCCs). However, these molecular markers cannot provide a convincing conclusion for all patients, due to limited numbers of alterations analyzed and the existence of intratumor heterogeneity 16. Next-generation sequencing (NGS) technology has greatly developed in recent years. Whole-genome sequencing (WGS) or whole-exome sequencing (WES) can be used to detect a large set of cancer variants for further analyses, including the clonal relationship and tumor heterogeneity of synchronous or metachronous multiple cancers 17-19. However, NGS-based assays have not yet been explored to distinguish primary LUSC from metastasis in patients with SCC of other sites, such as the head and neck, esophagus, or cervix.

In this study, we performed WES on tumor tissues from 12 patients with double SCCs of the esophagus and lung, which were considered LM according to the clinicopathological criteria. We aimed to assess the feasibility of evaluating the clonal relationships of double SCCs in the esophagus and lung using WES.

## Materials and Methods

### Patients

A total of 14 patients (P1-P14), including 12 with double SCCs of the esophagus and lung, and 2 ESCC patients with paired primary and non-lung hematogenous metastatic tumors (1 ESCC patient with brain and esophageal regional LN metastases, and 1 ESCC patient with kidney parenchymal metastasis), were recruited in our study (Table 1 and Table S1). Resection was performed for each tumor lesion (including SCCs in the esophagus, lung, brain and kidney) at the National Cancer Center/National Clinical Research Center for Cancer/Cancer Hospital, Chinese Academy of Medical Sciences and Peking Union Medical College. Of the 14 patients, 10 had synchronous double SCCs (interval time ≤ 6 months), and 4 had metachronous double SCCs (interval time > 6 months). None of these patients underwent preoperative chemotherapy or radiation therapy. Pathological diagnoses were independently made by two experienced pathologists based on hematoxylin and eosin (HE) stained slides (Figure S1). The study was approved by the Institute Review Board of the National Cancer Center/National Clinical Research Center for Cancer/Cancer Hospital, Chinese Academy of Medical Sciences and Peking Union Medical College. Informed consent was obtained from all of the patients. Follow-up started at the time of pulmonary surgery (or kidney surgery for P5 and brain surgery for P8).

### Immunohistochemical assay (IHC)

IHC was performed to explore the protein expression of p53 in the tumors of esophagus (T), LN and lung (D) from patients with double SCCs of the esophagus and lung. Briefly, the tumor tissue sections were stained in an autostainer (Autostainer Link 48, Dako, Denmark) with the antibody against p53 (DO-7, Dako). We scored p53 expression in three groups: weak or patchy (wild type), complete loss (nonsense, frameshift or splice-site mutation type), and diffuse and strong (missense mutation type). The latter two groups were considered aberrant p53 expression 20.

### Samples and DNA extraction

All HE slides were reviewed by two experienced pathologists, and formalin-fixed and paraffin-embedded (FFPE) tumor samples from the esophagus, lung (or kidney for P5 and brain for P8) and LN (except for P5 and P7) with appropriate tumor cellularity (>70%) were sectioned from selected blocks. Genomic DNA was extracted using QIAamp DNA FFPE Tissue Kits (Qiagen, Duesseldorf, Germany) according to the manufacturer's instructions. DNA quantity was measured using a Qubit 2.0 Fluorometer (Thermo Fisher Scientific, Carlsbad, CA, USA), and DNA quality was evaluated by 1% agarose gel electrophoresis. A total of 40 tumor samples from 14 patients were subjected to WES, and normal paracancerous tissues were used as controls to identify somatic alterations.

### WES

The qualified genomic DNA was randomly fragmented by Covaris to generate 180 to 280 bp DNA fragments. The DNA fragments were ligated with adapters and amplified by ligation-mediated PCR. The PCR products were purified and hybridized to the SureSelect biotinylated RNA library (Agilent Technologies, Santa Clara, CA, USA) for enrichment. Next, the captured PCR products were assessed using an Agilent 2100 Bioanalyzer and quantitative PCR. Finally, the qualified captured library of each sample was mixed and then processed with an IlluminaHiSeq 2000 System. The raw sequence data reported in this paper have been deposited in the Genome Sequence Archive 21 in National Genomics Data Center 22, Beijing Institute of Genomics (China National Center for Bioinformation), Chinese Academy of Sciences, under accession number HRA000254, and they are publicly accessible at http://bigd.big.ac.cn/gsa-human (https://bigd.big.ac.cn/gsa-human/browse/HRA000254).

### Sequence alignment and variant annotation

Raw data were filtered to remove low-quality reads and sequencing adapters, and to produce clean data**.** All of the clean data were then aligned to the human reference genome (GRCh37/hg19) using Burrows-Wheeler Aligner (BWA v0.7.15) software 23. Local realignment around indels was performed using IndelRealigner and RealignerTargetCreator in GATK (v1.0.6076) according to GATK Best Practice 24, with duplicate reads removed by Picard tools. After base quality score recalibration with GATK, somatic single nucleotide variations (SNVs) were detected by MuTect 25. Then, we used our in-house pipeline to identify the high-confidence variants with the major criteria as follows: coverage for both normal and tumor samples ≥ 10; the variant allele fraction (VAF) > 10% in tumor samples and < 2% in normal samples; and coverage for the variant allele ≥ 3. Somatic indels were predicted by the GATK SomaticIndelDetector with default parameters. Then we developed an in-house pipeline to obtain high-confidence somatic indels, which included the following steps: i) the combined normal and tumor bam files were reused to perform local realignment; ii) germline indels were filtered to obtain high-confidence indels; iii) normal coverage and tumor coverage ≥ 10; and iv) high-confidence somatic SNVs and indels were annotated using SnpEff (version 4.0).

### Clonal and evolution analyses

Phylogenetic analyses were conducted with nonsynonymous somatic mutated genes. Branch and trunk lengths were proportional to the number of nonsynonymous mutated genes. Moreover, we analyzed the B-allele frequency (BAF) distribution of mutations in the T, D and LN tumors of each patient.

### Identification of significantly mutated genes (SMGs)

SMGs were identified using the MutSigCV method, which quantified the significance of non-silent mutations in genes with background mutation rates estimated by silent mutations. *TP53*, *RPL5* and *MUC16* were identified as SMGs. We also performed an analysis focusing on the mutations in our samples that had been previously reported in the COSMIC (Catalogue of Somatic Mutations in Cancer) database and obtained 17 candidate genes (mutations found in at least two patients).

### Mutation spectra and mutation signature analyses

Mutation spectra were analyzed in each sample, and mutation spectral plots were drawn to investigate the relationship among T, D and LN tumors in the same patient. Mutation signatures were submitted to a multiple regression method using the deconstructSigs 26 package in R software. Signatures 1-30 were based on the Wellcome Trust Sanger Institute COSMIC Mutational Signature Framework. The contribution of each signature for each tumor was statistically quantified.

### Focal somatic copy-number alterations (SCNAs)

SCNAs were detected by GATK for each tumor sample. To infer recurrently amplified or deleted genomic regions, we re-implemented the GISTICalgorithm32 using copy numbers in 1-kb windows, instead of SNP array probes as markers. G-scores were calculated for altered genomic regions based on the frequency and amplitude of amplification and deletion. A significant SCNA region was defined as corresponding to a P-value threshold of 0.05 from permutation-derived null distribution and peak regions for further analysis. For each region, we evaluated the copy number aberrations level of each sample. The region with a positive log2 copy ratio was determined for amplification, and the negative region was deleted. For amplification regions, log2 copy ratios greater than 0.9 were determined to be high-level copy number aberrations, and those with ratios less than 0.9 and greater than 0.1 were determined to be low-level aberrations. For deletion regions, a log2 copy ratio less than -1.3 was determined to be a high-level copy number aberration, and those with ratios greater than -1.3 and less than -0.1 were determined to be low-level aberrations. The cosine similarity was calculated based on copy aberrations level classifications of SCNAs on focal amplification and deletion regions.

### Statistical analyses

The chi-square test was used to assess the differences in mutation spectra among the T, D and LN tumors. We also compared differences between the T tumors in our cohort and 96 ESCC samples from the Cancer Genome Atlas (TCGA) database and differences between the D tumors that shared no mutations with T tumors in our cohort identified by WES and 177 LUSC samples from the TCGA using the chi-square test. A two-sided P-value < 0.05 was considered statistically significant.

## Results

### Sequencing of paired synchronous/metachronous double SCCs of the esophagus and lung

To investigate the feasibility of distinguishing LM from SPT, we selected 35 tumors, including 12 esophageal tumors (T), 12 lung tumors (D) and 11 LN metastases (LN), from 12 patients with double SCCs of the esophagus and lung for molecular analysis (Figure S2). All 12 patients were diagnosed with LM according to the following pathological information: (i) peripheral location; (ii) histological similarity between double SCCs in the esophagus and lung; and (iii) the absence of SCC *in situ* or severe dysplasia adjacent to the lung tumor. All but one of these patients had LN metastases (except for P7) (Figure S1). The LN metastatic tumor of each patient (except for P7) was sequenced as an internal mimic control for LM. We also sequenced the whole exomes of 5 tumors (P5, 1 esophageal tumor and 1 paired metastatic kidney parenchymal tumor; P8, 1 esophageal tumor, 1 metastatic brain tumor and 1 LN metastasis) from 2 patients with non-lung hematogenous metastatic ESCCs, which were used as external mimic controls for LM (Table 1 and Table S1). The average coverage of all of the samples was approximately 149×. Approximately 93.2% of the targeted bases in each sample had at least 10× depth (Table S2). Strict data quality control was performed for each sample to exclude sequence artifacts of the FFPE samples. Finally, a median of 71 somatic mutations per tumor was identified (ranging from 13 to 237) (Table S3).

### Shared somatic mutations in double SCCs of the esophagus and lung

**Figure 1:**
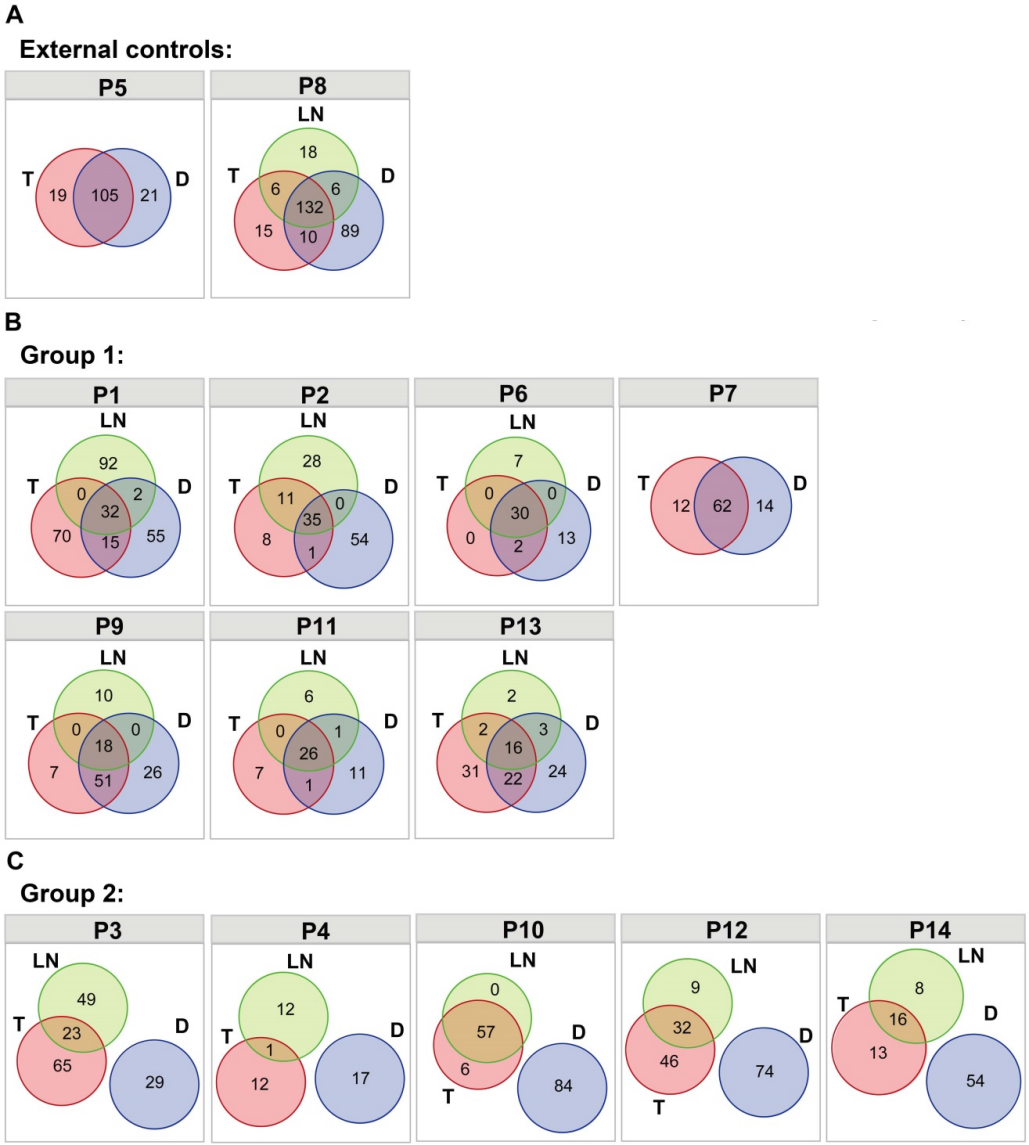
The distribution of nonsynonymous somatic mutations among different tumors. (A) Venn diagrams showing the distribution of nonsynonymous somatic mutations among different tumors within the same patient in P5 and P8 (external controls). (B) Venn diagrams showing the distribution of nonsynonymous somatic mutations among different tumors within the same patient in P1, P2, P6, P7, P9, P11 and P13 (group 1). (C) Venn diagrams showing the distribution of nonsynonymous somatic mutations among different tumors within the same patient in P3, P4, P10, P12 and P14 (group 2).

Somatic nonsynonymous mutations were analyzed in different tumors from the same patients (Table S4), and classified as trunk (T&LN&D shared mutations in P1-P4, P6, and P8-P14; T&D shared mutations in P5 and P7), branch (T&D shared mutations, T&LN shared mutations and D&LN shared mutations in P1-P4, P6, and P8-P14) and private mutations. The two primary and metastatic tumor pairs were used as external controls (P5 and P8). As shown in Figure 1A, trunk mutations accounted for 72.4% of all mutations in P5 and 47.8% of all mutations in P8. Similarly, diverse extents of trunk mutations (12.0%-70.5%) were detected in P1, P2, P6, P7, P9, P11 and P13, which were classified as group 1 (Figure 1B). These data suggest the possibility of LM tumor pairs in these patients. However, neither trunk mutations nor T&D shared branch mutations were observed in P3, P4, P10, P12 and P14 (group 2) (Figure 1C), indicating that the double SCCs of the esophagus and lung in these patients were likely independent tumors. In addition, LN tumors shared mutations with T tumors but not with D tumors in these patients, suggesting that the LN tumors derived from SCCs of the esophagus were independent of the lung lesions in these patients.

Furthermore, overlapping somatic nonsynonymous mutations between different tumors from different patients were also evaluated to explore whether the shared mutations (trunk and branch mutations) in these samples could occur by chance. As shown in Figure S3, no shared mutations were observed between different patients. These data indicate that shared mutations between different tumors within one patient strongly suggest evidence of clonal relatedness rather than coincidence.

### Clonal relatedness analyses in double SCCs of the esophagus and lung

**Figure 2:**
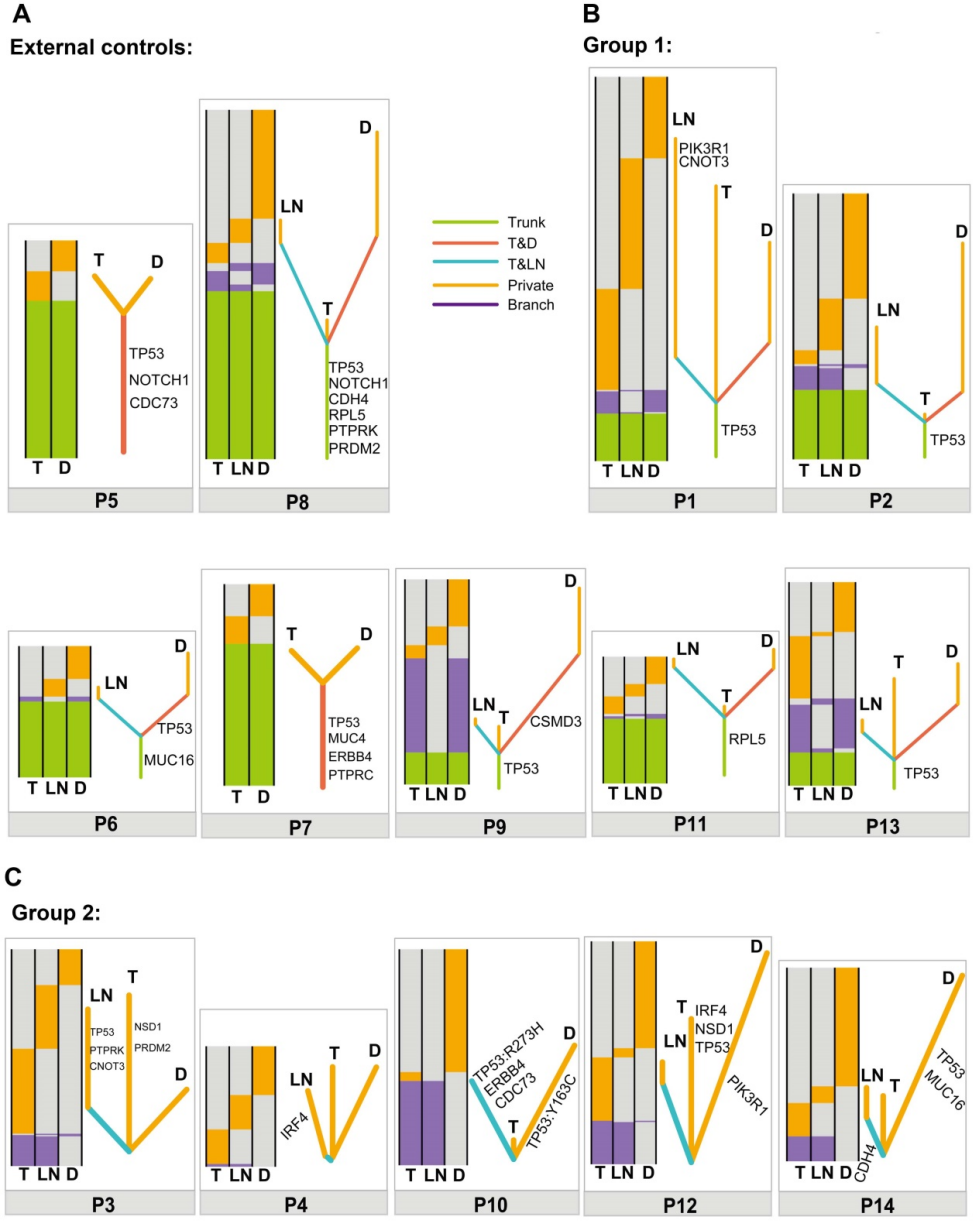
Relationship of different tumors from one patient based on somatic mutated genes. (A) Heatmaps (left) and phylogenetic trees (right) showing the relationship of different tumors from one patient based on somatic mutated genes in P5 and P8 (external controls). (B) Heatmaps (left) and phylogenetic trees (right) showing the relationship of different tumors from one patient based on somatic mutated genes in P1, P2, P6, P7, P9, P11 and P13 (group 1). (C) Heatmaps (left) and phylogenetic trees (right) showing the relationship of different tumors from one patient based on somatic mutated genes in P3, P4, P10, P12 and P14 (group 2). Trunk, branch and private mutations are illustrated in the heatmaps.

To further determine the clonal relatedness of double SCCs in the esophagus and lung, a phylogenetic tree was constructed using somatic mutated genes in each patient. Similar to the results in the external controls (Figure 2A), the results in Figure 2B showed that different lengths of trunk genes were observed in group 1 (P1, P2, P6, P7, P9, P11 and P13), indicating a monoclonal origin. However, no length of trunk or T&D shared branch genes was observed between T and D tumors in group 2 (P3, P4, P10, P12 and P14) (Figure 2C). These data suggest that double SCCs of the esophagus and lung in these patients likely have multiple independent origins.

Moreover, the BAF profiles of T, D and LN tumors from the same patient were compared by BAF distribution. The results revealed that shared mutation clusters were observed in the external controls and group 1 (Figure S4A and S4B), suggesting a common clonal origin of different tumors in a single patient. In group 2, distinct mutation clusters were detected between T and D (Figure S4C), suggesting independent origins between T and D tumors in each patient.

### SMGs in double SCCs of the esophagus and lung

**Figure 3:**
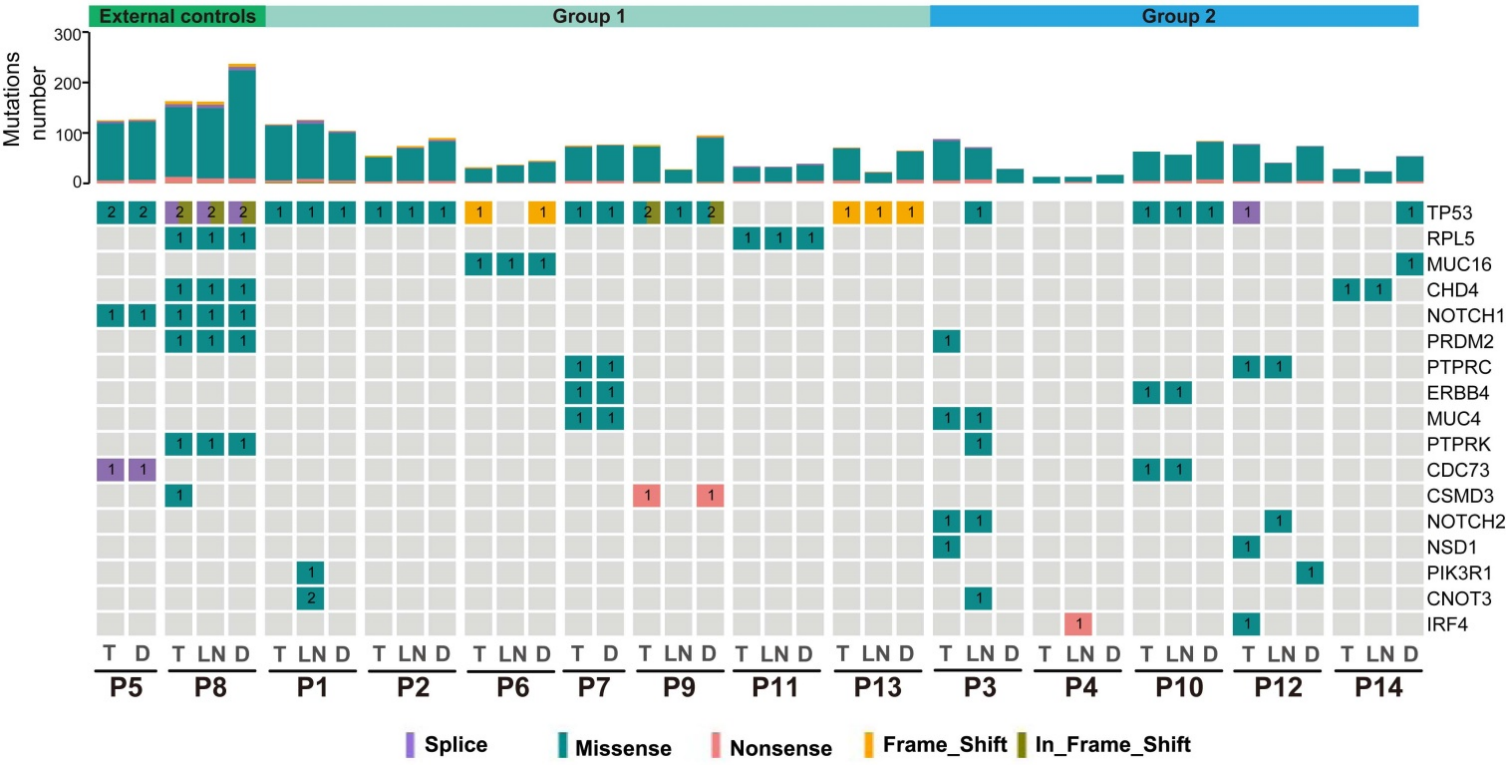
Summary of significantly mutated genes (SMGs) in the 14 ESCC patients. Commonly mutated genes in at least two patients are listed. Top: total point mutations and indels. Bottom: presence or absence (gray) of SMGs in each tumor.

The identification of SMGs is essential to discover candidate driver genes for tumor development 27. In total, we identified 17 SMGs, and each gene was mutated in at least 2 patients. *TP53* was the most common SMG in these tumors (27/40, 67.5%). Moreover, 1 to 7 shared SMGs were identified between T and D tumors in P1, P2, P5-P9, P11 and P13 (external controls and group 1). Shared SMGs were observed between T and LN tumors in P3, P12 and P14, but no shared SMGs were detected between T and D tumors in these patients. In P4, an *IRF4* mutation was detected in the LN tumor but not in the T or D tumor (Figure 3). In P10, two different *TP53* mutations were observed in T&LN (*TP53*, c.818G>A, p.Arg273His) and D (*TP53*, c.488A>G, p.Tyr163Cys) tumors.

### Mutation spectra and mutation signatures in double SCCs of the esophagus and lung

**Figure 4:**
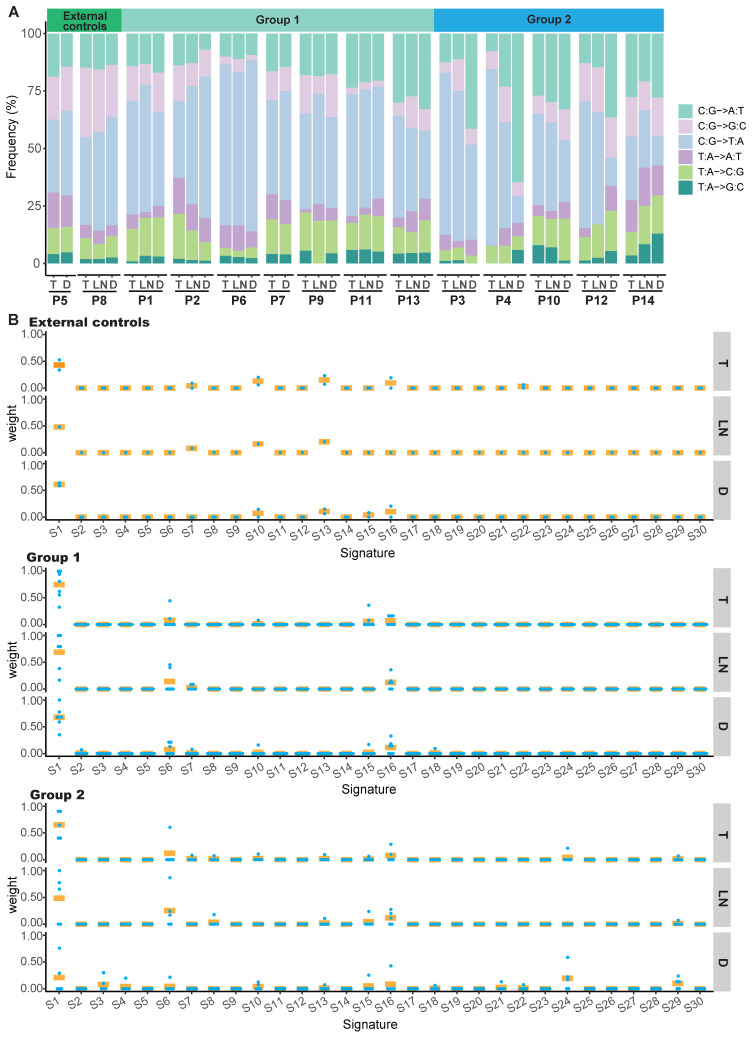
Mutation spectra and contributions of signatures in patients with double SCCs. (A) Mutation spectra of all tumors in the external controls, group 1 and group 2. (B) The contribution of signatures to T, LN and D tumors in the external controls, group 1 and group 2. S1-30 are the signatures in the COSMIC database. Each dot represents one tumor and bars represent mean values.

We identified a predominance of the C>T/G>A transition in the T, LN or D tumors of the external controls and group 1 (Figure S5A and S5B, Figure S6A and S6B). However, we identified a predominance of the C>T/G>A transition in T and LN tumors, but a predominance of the C>A/G>T transition in D tumors of group 2 (P<0.001) (Figure S5C and Figure S6C). The mutation spectra of T tumors in our cohort were similar to those of 96 ESCC samples from TCGA (Figure S6D), whereas no statistically significant difference was found in the mutation spectra between D tumors in group 2 and 177 LUSC samples from TCGA (Figure S6E). Moreover, the mutation spectra between T and D tumors from the same patient were nearly identical in the external controls and group 1, further supporting the monoclonality of T and D tumors in these patients (Figure 4A, Figure S7A and S7B). The mutation spectra between T and D tumors were discordant in group 2. However, similar mutation spectra were observed between T and LN tumors in these cases (Figure 4A and Figure S7C). These data suggest that the T and D tumors in group 2 were from multifocal origins and the LN tumors were derived from T tumors.

The overall mutation signatures were similar among T, LN and D tumors of the external controls and group 1 with very strong enrichment of signature 1 (associated with age). In group 2, although strong enrichment of signature 1 was observed in T and LN tumors, the contribution of signature 1 in D tumors was decreased as compared to other tumors (Figure 4B and Figure S8). Signature 29 (associated with tobacco chewing), signature 24 (associated with aflatoxin), signature 3 (associated with failure of DNA double-strand break-repair by homologous recombination), signature 6 (associated with defective DNA mismatch repair) and signature 16 (associated with an unknown aetiology) were observed in the D tumor of group 2.

### Focal SCNAs in double SCCs of the esophagus and lung

**Figure 5:**
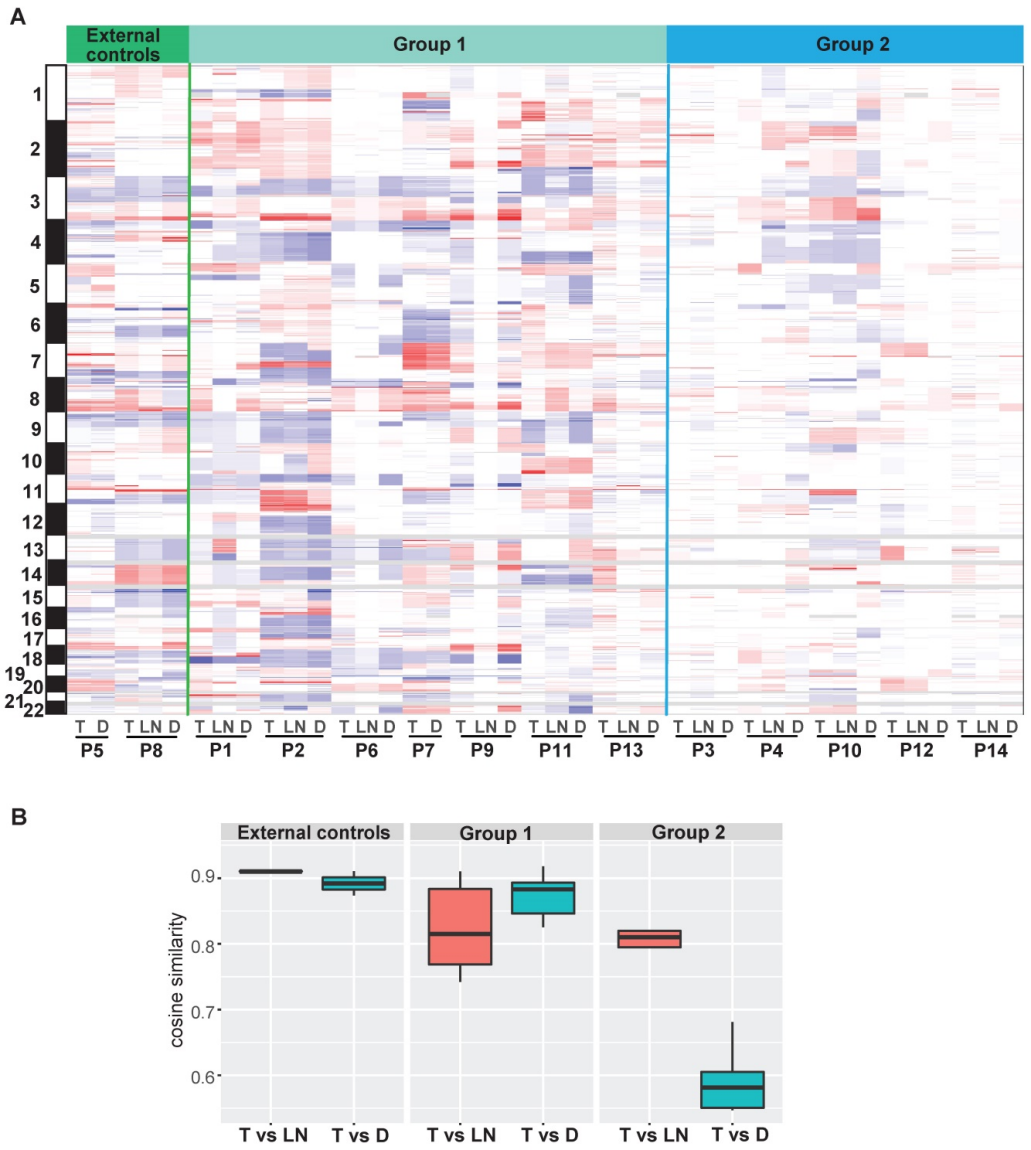
The profiles of focal somatic copy-number alterations (SCNAs). (A) Distribution of focal SCNAs in all tumors. Red: SCNA gain; blue: SCNA loss. (B) The distribution of cosine similarity between T and LN tumors, as well as between T and D tumors in each group.

Focal SCNA segments, ranging from 56 to 116, were identified in each tumor (Table S5). A number of ubiquitous SCNAs were detected between T and D tumors in the external controls and group 1. In group 2, several ubiquitous SCNAs were observed between T and LN tumors, whereas the SCNA profiles were different between T and D tumors in the same patient (Figure 5A and Figure S9). Moreover, the distribution of cosine similarity between T and D was significantly different from that between T and LN in group 2. The median of cosine similarity between T and D in group 2 was less than 0.6, while that of the other groups was greater than 0.8 (Figure 5B).

### Comparison of molecular discrimination with pathological diagnosis

**Figure 6:**
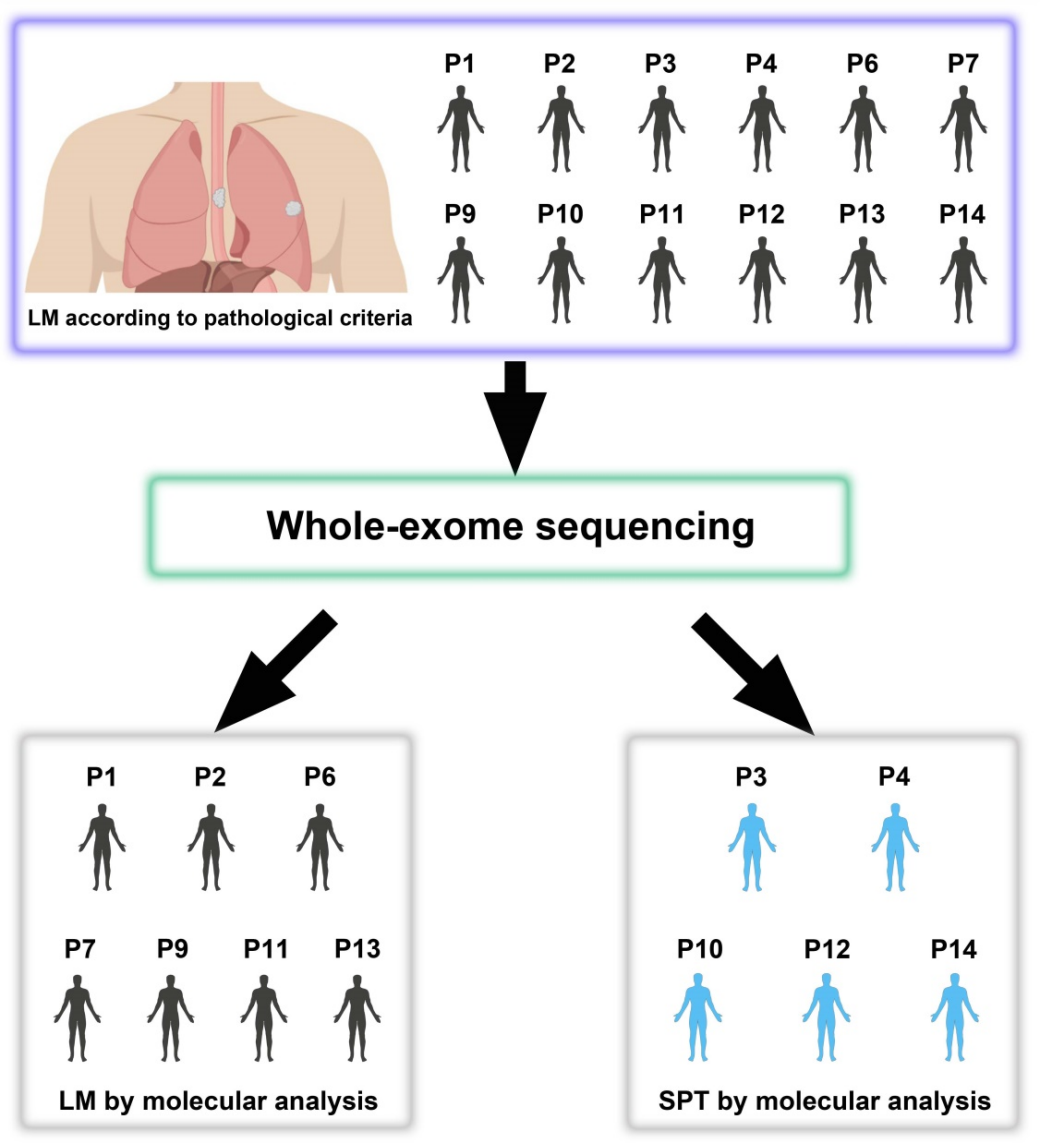
Twelve patients with double SCCs of the esophagus and lung diagnosed as lung metastasis (LM) using pathological information are subjected to WES, but 5 patients (41.7%) are considered second primary tumors (SPTs) according to the WES-based molecular analysis.

Based on the clonal evolution theory, primary and metastatic tumors are derived from a common clone with identical or similar genetic profiles; in contrast, distinct genomic profiles most likely imply independent clonal origins. For the 12 patients with double SCCs of the esophagus and lung, inconsistent results between pathological and molecular diagnoses were found in 5 patients (41.7%) (Figure 6 and Table 1). All 5 of the patients (P3, P4, P10, P12 and P14) were diagnosed with LM using pathological information. However, these cases were considered SPT according to our WES results, since then T and D tumors were suggested to arise from independent clones. Interestingly, all 5 of these patients showed a relatively good prognosis after pulmonary surgery (the survival time ranged from 12 to 90 months). However, given the small sample size in this study, it is difficult to draw any conclusion in terms of prognosis. Moreover, p53 expression was detected using IHC in the T, LN and D tumors of the 12 patients with double SCCs of the esophagus and lung. In P14, complete loss of p53 expression was observed in T and LN tumors, but diffuse and strong expression of p53 was observed in the D tumor (Figure S10), further supporting the results of WES-based molecular diagnosis. However, the same p53 immunostaining status was observed between T and D tumors in the remaining 11 patients, indicating the limitations of p53 IHC for use in distinguishing SPT from LM (Table S1).

## Discussion

Patients with double SCCs of the esophagus and lung can be diagnosed with LM or SPT. Clinically, if LM is diagnosed, chemotherapy is recommended, and the prognosis is usually poor due to the late stage. However, chemotherapy is not necessary for SPT in early stages, and the prognosis is usually good. Thus, dependable markers for the differential diagnosis of LM and SPT are urgently required. In this study, we explored the clonal relationship of different tumors within the same patient in 12 patients with double SCCs of the esophagus and lung using WES. Two primary and non-lung hematogenous metastatic ESCC pairs were interrogated as external controls, and the esophageal regional LN metastatic tumor in each case was sequenced as an internal control. Genomic profiles, including somatic nonsynonymous point mutations, BAFs, SMGs, mutation spectra, mutation signature and focal SCNAs, were compared between esophageal and lung lesions in each patient. A number of similar genomic alterations were observed in 7 tumor pairs, suggesting a common clonal origin. Nevertheless, distinct genomic profiles were observed in the other 5 tumor pairs, indicating multiple independent origins.

Currently, distinguishing SPT from LM in patients with double SCCs of the esophagus and lung mainly relies on clinicopathological criteria 28. For LM, computed tomography (CT) image of lung tumor usually shows a peripheral location and multiple nodules with a round shape and smooth contour, and pathological examination shows histological similarity between lung and esophageal tumors. For SPT, CT imaging of the lung tumor usually exhibits a central location and solitary nodules with a lobulated sign or irregular shape, and pathological examination sometimes shows SCC *in situ* or severe dysplasia adjacent to the lung tumor. However, there is no consensus regarding LM/SPT discrimination. In this study, we retrospectively examined 12 patients with double SCCs of the esophagus and lung that were considered LM by two independent pathologists. The LM diagnosis was made on the basis of the information regarding peripheral location, histological similarity and the absence of SCC *in situ* or severe dysplasia. Moreover, LN metastases, poor or basaloid differentiation, and lymphovascular invasion, which were observed in most patients, were considered indicators of the onset of metastases (Table S1). However, solitary lung SCC, the absence of systemic metastases in other organs (P1-P4, P6, P7, P9-P14), and an interval longer than two years (P1 and P13) in these patients did not support the diagnosis of LM. Therefore, the diagnoses in our cohort were conflicting according to the clinicopathological information.

Based on the cancer evolution theory, LM tumor pairs arise from the same progenitor cells and are clonally related. Conversely, SPT pairs arise from independent precursors and show completely discordant genomic profiles. To accurately diagnose double SCCs of the lung and other sites, several molecular biomarkers have been investigated to determine the clonal relationships of different tumors within one patient. For example, Geurts et al. have reported that *TP53* mutation and LOH analysis may be helpful for the differential diagnosis of secondary lung tumors in patients with head and neck squamous cell carcinoma (HNSCC) 15. Bishop et al. have reported that HPV typing may be a useful method to distinguish primary tumors from metastatic SCCs of the lung 29. Nevertheless, it is difficult to achieve a definitive differential diagnosis for all patients using these markers, since only a small proportion of alterations have been detected in these studies. Negative results or discordant alterations because of intratumor heterogeneity can influence accurate discrimination, even leading to a false diagnosis 30, 31. Moreover, there are few reports of molecular discrimination between LM and SPTs in patients with double SCCs of the esophagus and lung. The development of NGS technology has made it possible to detect multiple genomic alterations in a single test. To distinguish multiple independent tumors from hematogenous metastases, clonality and heterogeneity have been analyzed using a large number of alterations detected by WES in multiple cancers. Most of these reports have focused on multiple tumors with the same organ, including multiple lung adenocarcinomas, multifocal hepatocellular carcinomas, and bilateral ovarian cancers 17, 32, 33. Some studies have also reported the utilization of the NGS assay for distinguishing metastasis from second primary cancers in patients with multiple tumors from different organs 34, 35. However, the application of WES to distinguish SPT from LM in patients with double SCCs of the esophagus and lung has not been described. In this study, we performed WES on samples from 12 patients that were pathologically diagnosed as LM. Based on the results obtained from the comparison of somatic nonsynonymous point mutations, BAFs, SMGs, mutation spectra, mutation signature and focal SCNAs between T and D tumors, we found that 7 tumor pairs were clonally related, whereas 5 tumor pairs were likely to have independent multiple clonal origins. Moreover, shared genomic profiles were observed between T and LN tumors (internal controls), as well as between 2 primary and metastatic ESCCs (external controls, P5 and P8), and no shared alterations were observed between two tumors from different individuals. In P14, IHC assay showed that different p53 immunostaining status was observed between T and D tumors. All of these data suggest that WES could be helpful for distinguishing SPT from LM.

Despite the small sample size, there were several interesting cases with distinctive clinicopathological features in our cohort. Usually, SPT is considered when the time interval is longer than two years. However, 2 of 3 patients (P1 and P13) were identified with LM, although the onset of their metachronous lung tumors was two years after the primary diagnosis. Moreover, the percentage of trunk mutations in P1 and P13 was relatively smaller than that in the other patients with LM (Figure S11), suggesting that longer time intervals between metachronous primary and metastatic tumors cause higher intratumor heterogeneity 36. For P4, mixed histological types were observed in T tumor, with approximately 95% basaloid SCC cells and 5% keratinizing SCC cells. However, only keratinizing SCC was observed in the LN tumor, which might have contributed to the only shared mutation between the T and LN tumors.

There were several limitations of our study. First, the number of patients with double SCCs in the esophagus and lung analyzed in this study was relatively small. Thus, our study should be regarded as providing descriptive observations, and future studies are needed to confirm our conclusions in a larger population. Second, the existence of intratumor heterogeneity in ESCC could affect the accurate molecular diagnosis of SPT 37, although the LN tumor in each case was also sequenced as the internal mimic control of LM. In addition, we used the archival FFPE blocks from surgically excised specimens with size of approximately 2 cm × 1 cm and high tumor cellularity (>70%), which might have minimized the effect of heterogeneity to some extent.

In conclusion, our study demonstrated the limitations of using pathological information only to distinguish SPT from LM. Comprehensive tumor genomic profiles could provide critical information for the accurate identification of LM/SPT in patients with double SCCs of the esophagus and lung. WES could be useful in understanding the clonal relationships of multiple SCCs.

## Supplementary Material

Supplementary figures and table 1.

Supplementary tables 2-5.

## Figures and Tables

**Table 1:** Clinicopathological features of 14 patients with double SCCs.

Patient ID	Gender	Age	Synchronous/metachronous	Tumor ID	Tumor location	Interval time (months)	Follow-up (months)	Pathological diagnosis	Molecular diagnosis
P1	Male	43	Metachronous	T	Esophagus	26	Deceased (10)	Metastasis	Metastasis
				LN	LN				
				D	Lung				
P2	Male	49	Synchronous	T	Esophagus	0	Deceased (3)	Metastasis	Metastasis
				LN	LN				
				D	Lung				
P3	Male	73	Synchronous	T	Esophagus	0	Deceased (35)	Metastasis	Primary
				LN	LN				
				D	Lung				
P4	Male	42	Metachronous	T	Esophagus	12	Alive (90)	Metastasis	Primary
				LN	LN				
				D	Lung				
P5	Male	54	Synchronous	T	Esophagus	4	Deceased (26)	Metastasis	Metastasis
				D	Kidney				
P6	Male	67	Synchronous	T	Esophagus	0	Deceased (23)	Metastasis	Metastasis
				LN	LN				
				D	Lung				
P7	Male	55	Synchronous	T	Esophagus	0	Deceased (25)	Metastasis	Metastasis
				D	Lung				
P8	Male	50	Synchronous	T	Esophagus	5	Alive (3)	Metastasis	Metastasis
				LN	LN				
				D	Brain				
P9	Male	56	Metachronous	T	Esophagus	18	Alive (53)	Metastasis	Metastasis
				LN	LN				
				D	Lung				
P10	Male	58	Synchronous	T	Esophagus	0	Alive (13)	Metastasis	Primary
				LN	LN				
				D	Lung				
P11	Male	80	Synchronous	T	Esophagus	0	Alive (3)	Metastasis	Metastasis
				LN	LN				
				D	Lung				
P12	Male	62	Synchronous	T	Esophagus	0	Alive (12)	Metastasis	Primary
				LN	LN				
				D	Lung				
P13	Male	49	Metachronous	T	Esophagus	36	Alive (41)	Metastasis	Metastasis
				LN	LN				
				D	Lung				
P14	Male	61	Synchronous	T	Esophagus	0	Deceased (20)	Metastasis	Primary
				LN	LN				
				D	Lung				

## Abbreviations

ESCC: esophageal squamous cell carcinoma
LUSC: lung squamous cell carcinoma
SCC: squamous cell carcinoma
LM: lung metastasis
SPT: second primary tumor
WES: whole-exome sequencing
T: esophageal tumor
LN: lymph node, or lymph node tumor (metastasis)
D: lung, brain, or kidney tumor
NGS: next-generation sequencing
HE: hematoxylin and eosin
FFPE: formalin-fixed and paraffin-embedded
SNV: single nucleotide variation
BAF: B-allele frequency
COSMIC: Catalogue of Somatic Mutations in Cancer
LOH: loss of heterozygosity
IHC: immunohistochemical assay
SMGs: significantly mutated genes
SNVs: somatic single nucleotide variations
VAF: variant allele fraction
TCGA: the Cancer Genome Atlas
