# Supplementary material for: Identification of second primary tumors from lung metastases in patients with esophageal squamous cell carcinoma using whole-exome sequencing
Source: Theranostics. 2020 Aug 25;10(23):10606–18. doi: 10.7150/thno.45311 (PMC7482800; doi:10.7150/thno.45311)
Supplement: Supplementary file 1 — Supplementary figures and table 1. [file thnov10p10606s1.pdf]

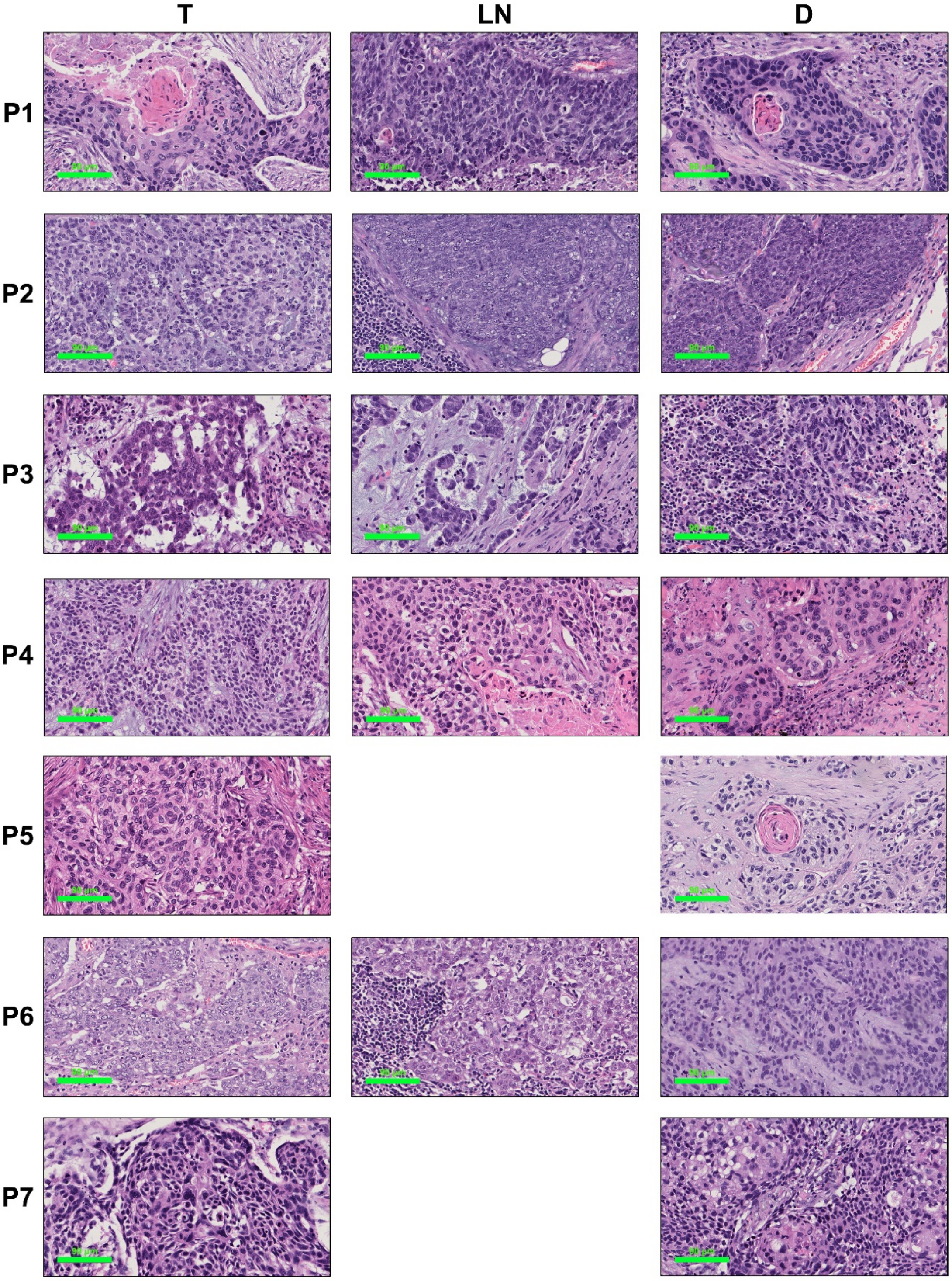
T
LN
D
P1
P2
P3
P4
P5
P6
P7

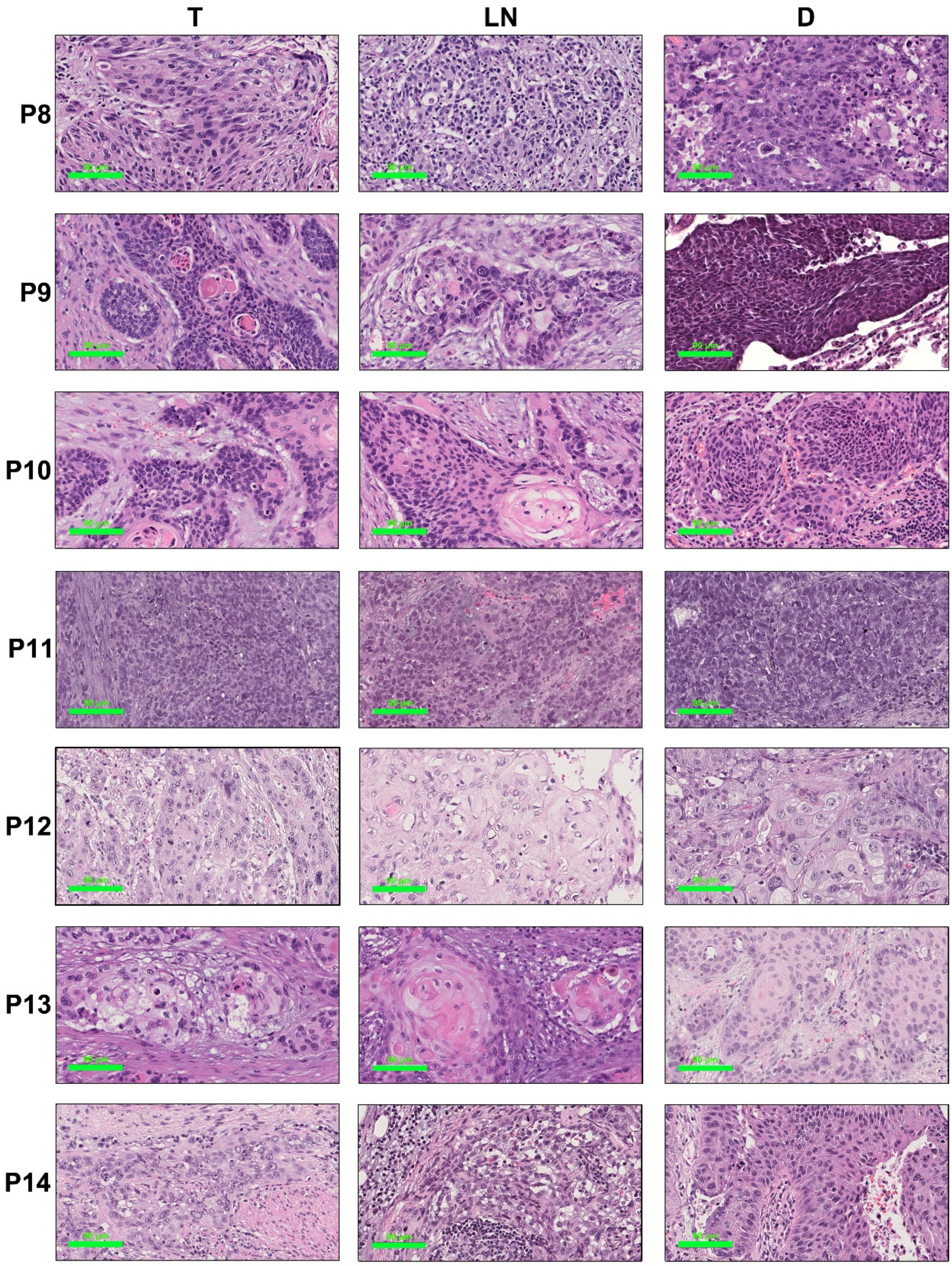

Figure S1. Representative histological morphology (magnification: 200×) of esophageal tumor (T), lung, brain, or kidney tumor (D) and lymph node metastasis (LN) from 14 patients. Bar, 90 µm.

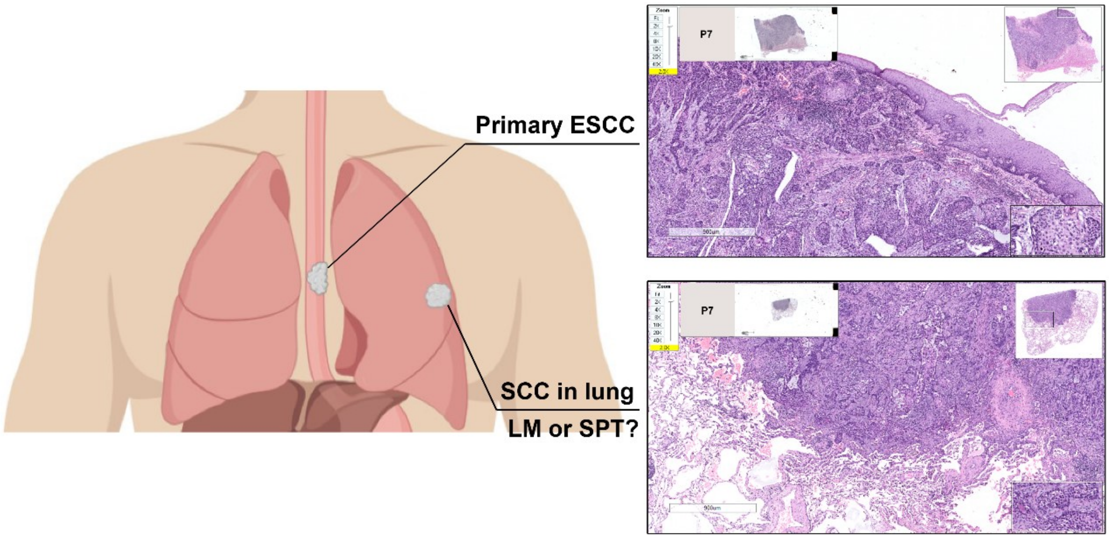

Figure S2. A schematic graph showing the anatomic sites and representative H&E staining of double esophageal and lung squamous cell carcinomas (SCCs), which is a clinical dilemma to accurately discriminate between lung metastasis (LM) and second primary tumors (SPTs). ESCC, esophageal squamous cell carcinoma. Bar, 900 µm.

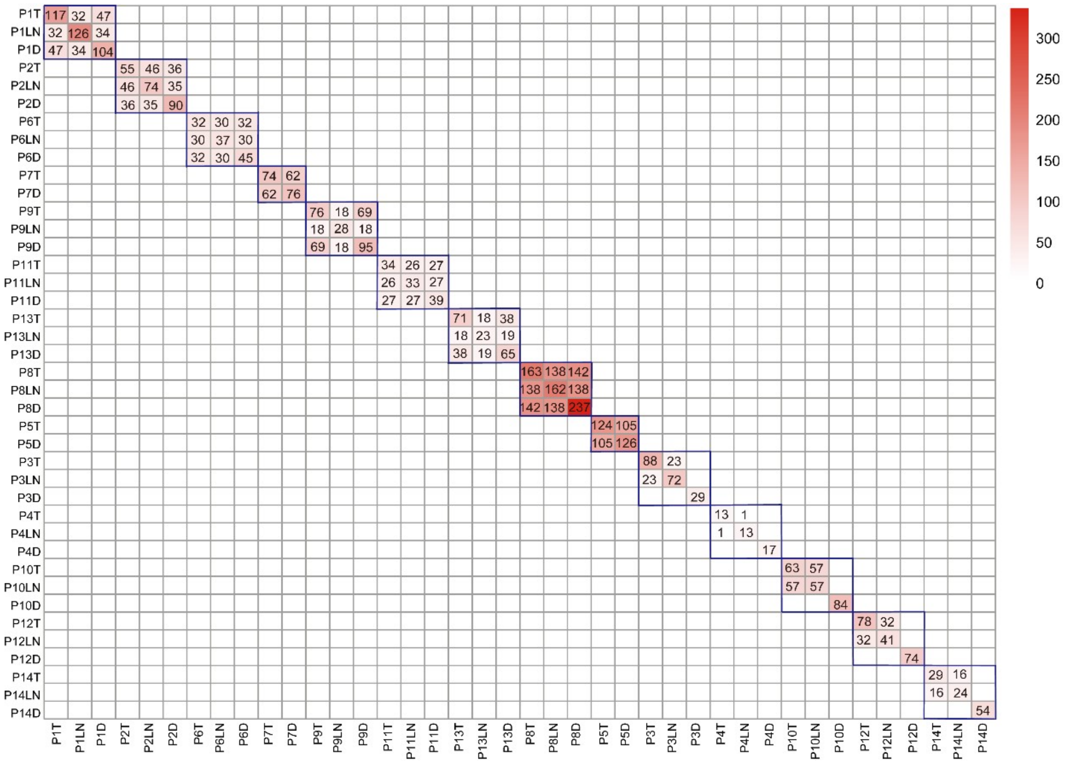

Figure S3. Heatmap of shared nonsynonymous somatic mutations between different tumors from 14 patients with double SCCs.

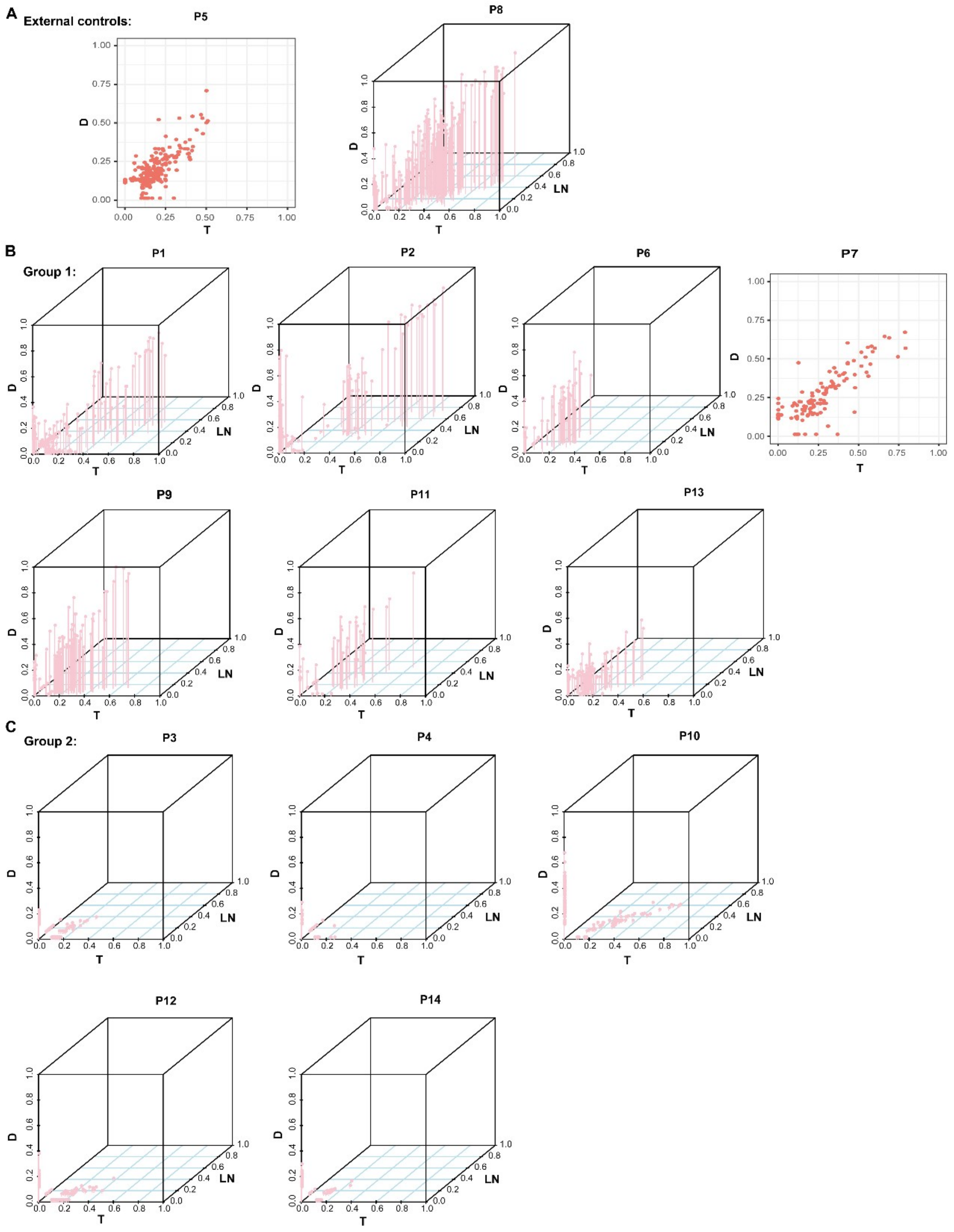

Figure S4. B-allele frequency (BAF) distribution showing the relationship of T, LN and D tumors within the same patient based on the BAFs in the (A) external controls, (B) group 1 and (C) group 2.

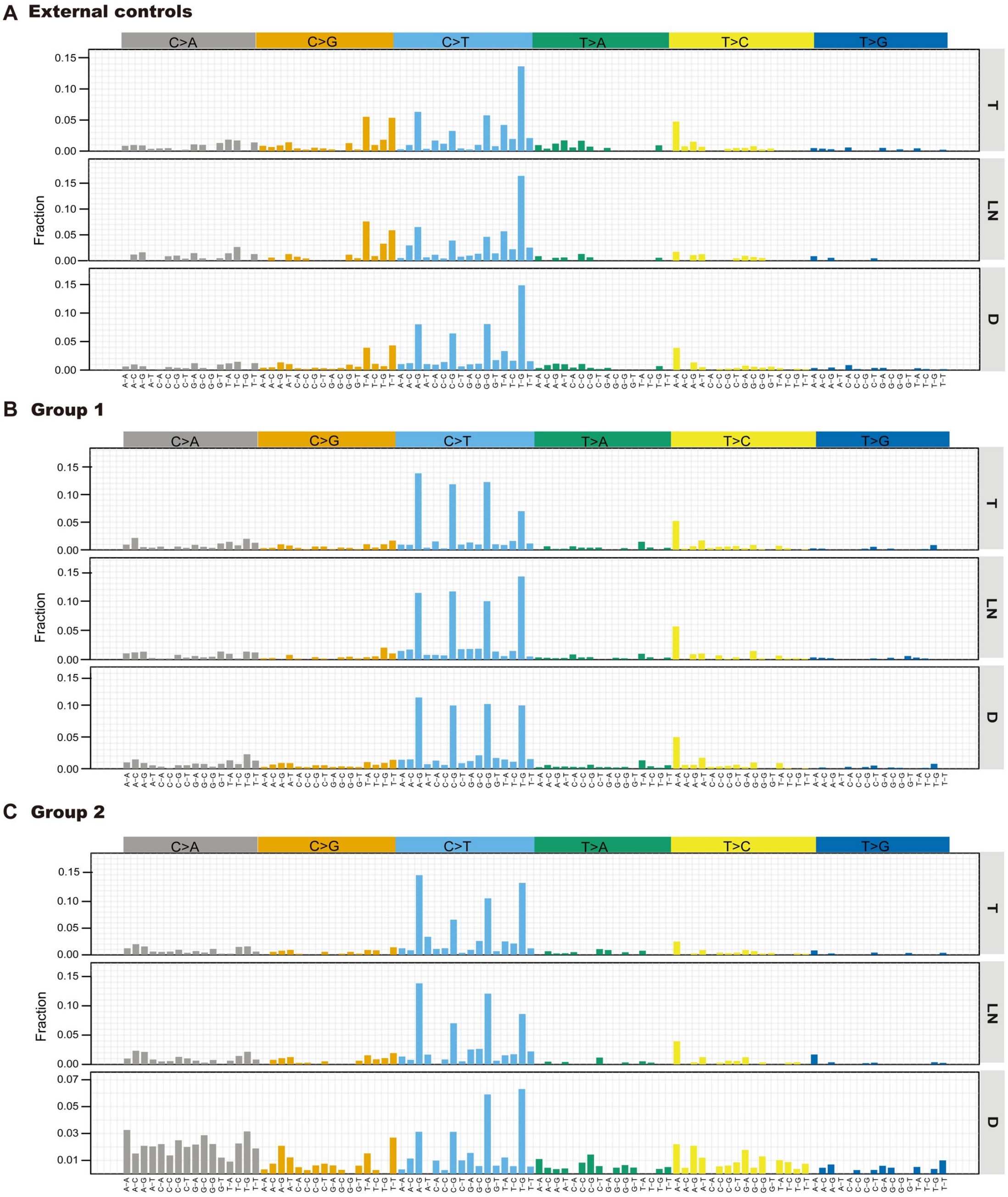

Figure S5. The 96 trinucleotide mutational spectra of T, LN and D tumors in the (A) external controls, (B) group 1 and (C) group 2.

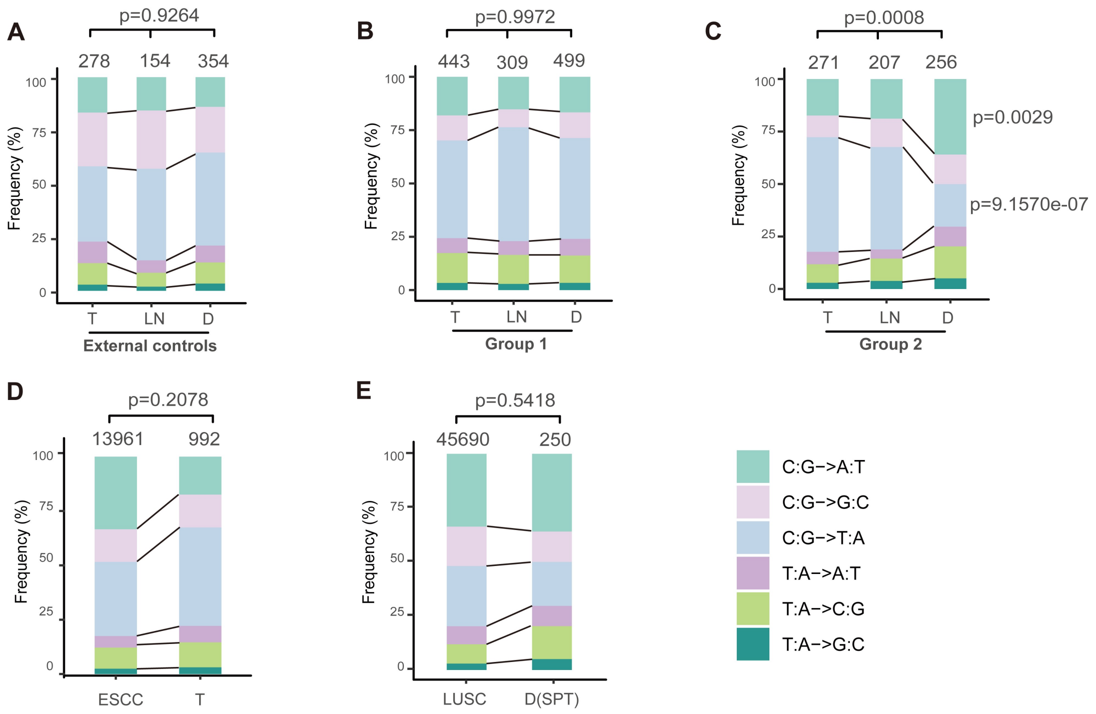

Figure S6. Analysis of mutation spectra in the external controls, group 1 and group 2. The fraction of mutations in T, LN and D tumors is displayed by a stacked bar plot. The differences between the spectra for mutations in T, LN and D tumors across the (A) external controls, (B) group 1 and (C) group 2, as well as (D) T tumors in our cohort and 96 esophageal squamous cell carcinoma (ESCC) samples from the TCGA, and (E) D tumors in the group 2 and 177 lung squamous cell carcinoma (LUSC) samples from the TCGA are analyzed. The number of mutations assessed is listed on top of each bar.

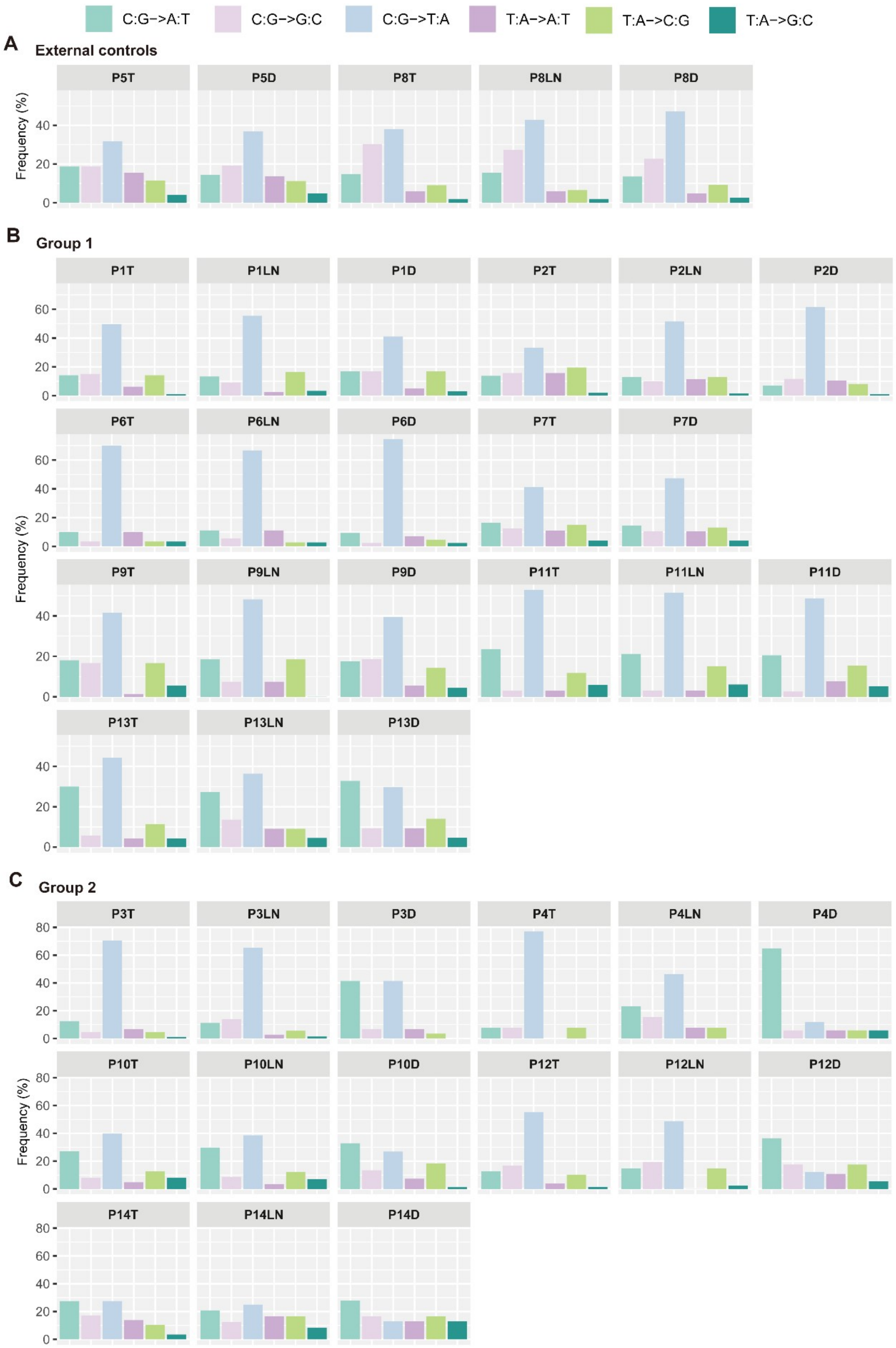

Figure S7. Mutation spectra of T, LN and D tumors in the (A) external controls, (B) group 1 and (C) group 2.

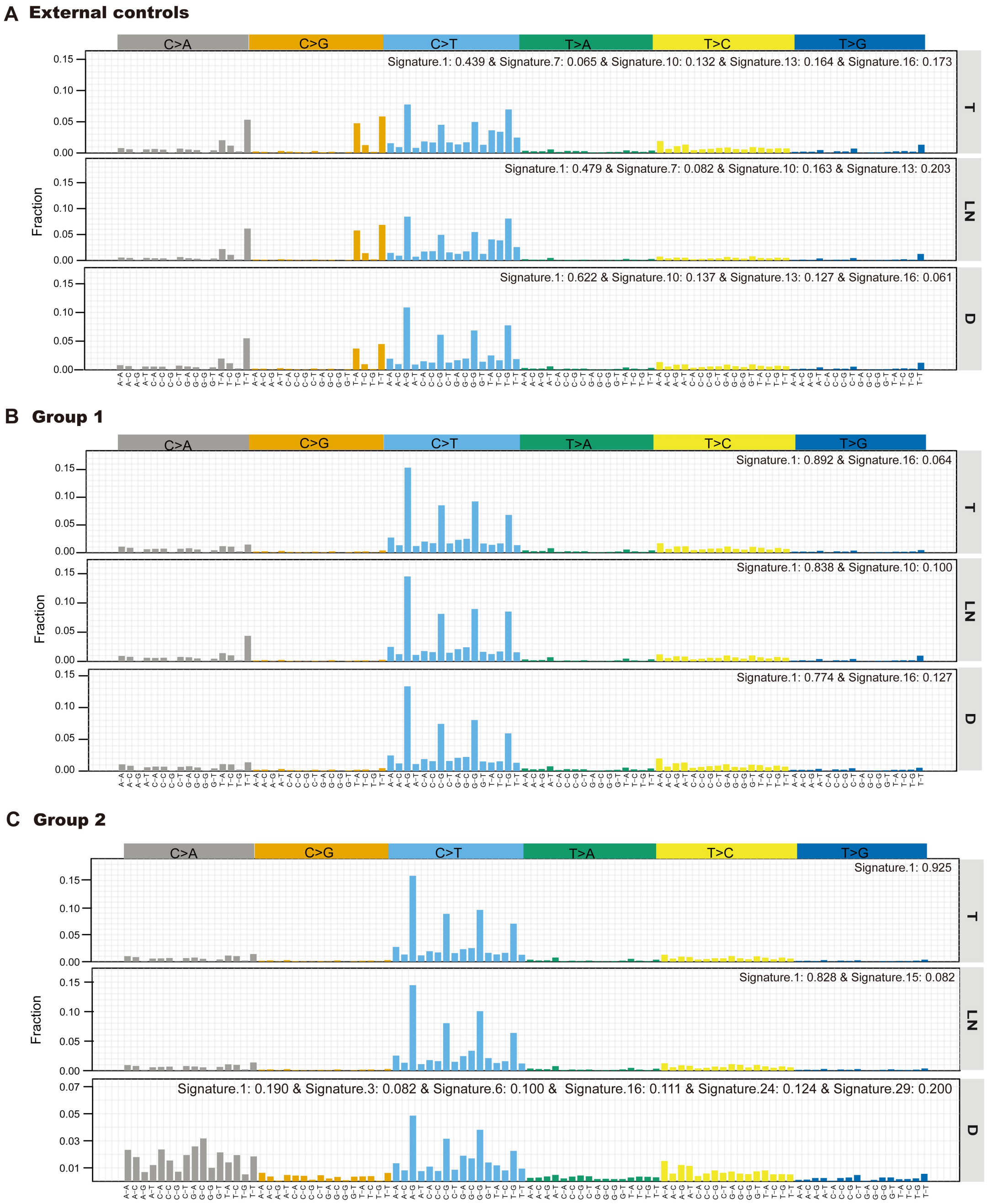

Figure S8. Mutational signatures of T, LN and D tumors in the (A) external controls, (B) group 1 and (C) group 2. The number in the upper right label is the contribution of signature for tumors.

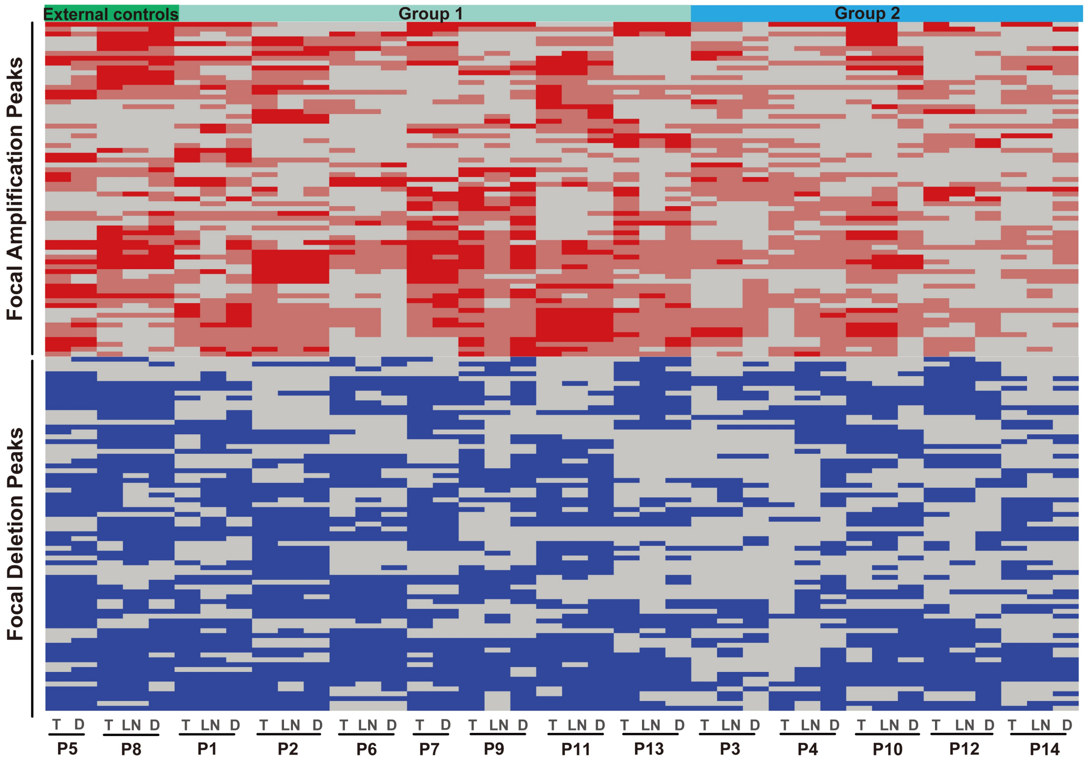

Figure S9. Heatmap showing the distribution of focal amplification and deletion peaks. Gray indicates the regions with no somatic copy-number alteration (SCNA) changes. Light red indicates the regions with SCNA changes, but not significant. Deep red indicates regions with significant SCNA changes. Blue indicates deletion regions.

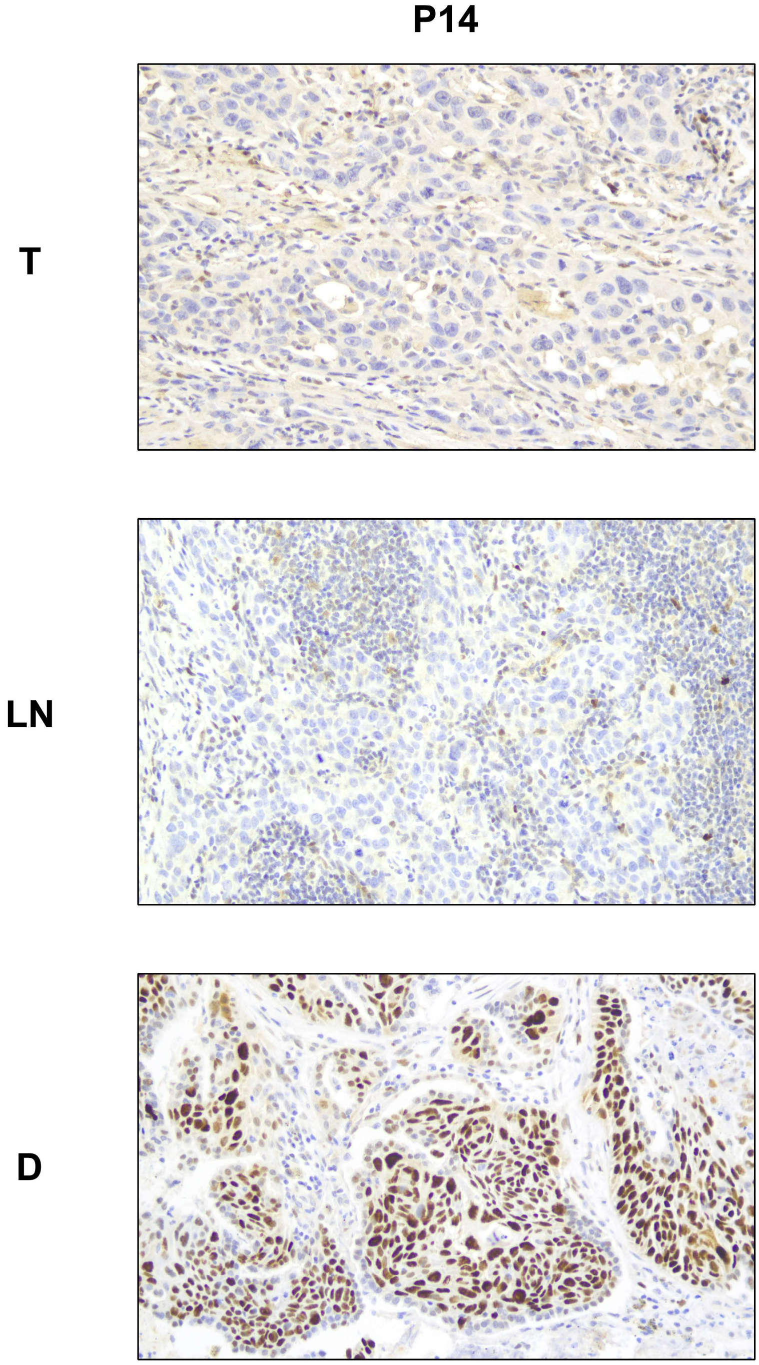

Figure S10. P53 immunostaining status in T, LN and D tumors of P14. Complete loss of p53 expression is observed in T and LN tumors, whereas diffuse and strong expression of p53 is observed in D tumor.

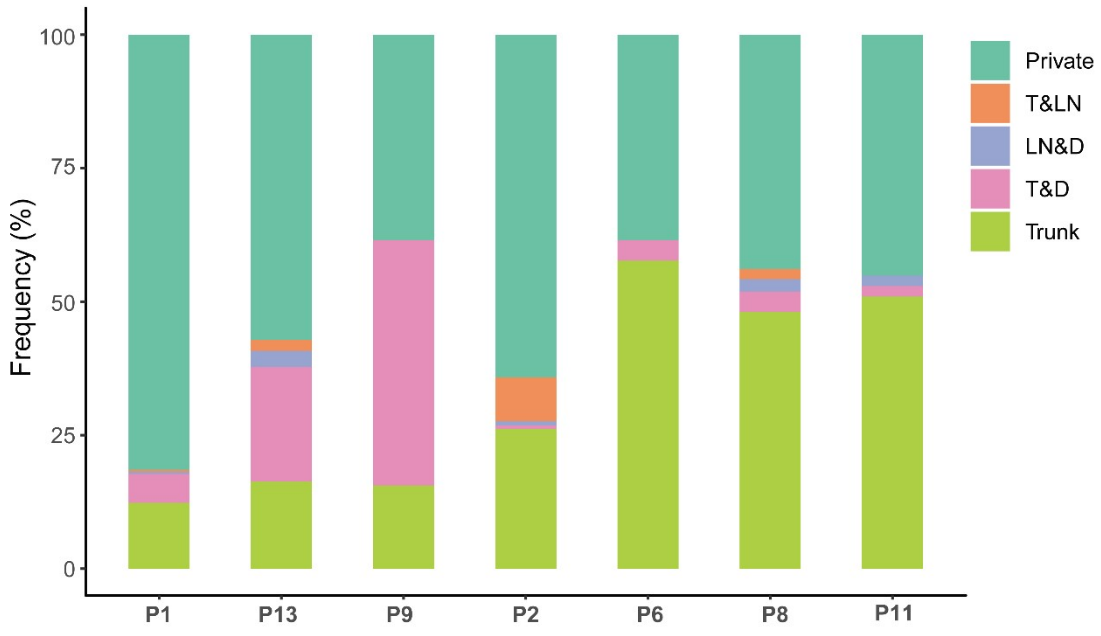

Figure S11. Percentage of truck, branch and private mutations in P1, P2, P6, P8, P9, P11 and P13.

Table S1. Clinicopathological features of 14 patients in our cohort.

Patient ID	Smoking	Drinking	Histopathology of ESCC					Histopathology of LUSC		
			Differentiation	pT status	pN status	LVI	P53 in T/LN tumors	Differentiation	Location	P53 in D tumor
P1	Yes	Yes	Moderate	T3	N3	No	Diffuse and strong	Moderate	LUL	Diffuse and strong
P2	Yes	Yes	Basaloid	T3	N1	Yes	Diffuse and strong	Basaloid	LUL	Diffuse and strong
P3	Yes	Never	Poor	T3	N1	No	Diffuse and strong	Poor	RUL	Diffuse and strong
P4	Yes	Yes	Moderate and basaloid	T2	N1	No	Complete loss	Moderate	RLL	Complete loss
P5	Yes	Yes	Poor	T2	N0	No	-	-	-	-
P6	Never	Never	Poor	T3	N2	No	Complete loss	Poor	RML	Complete loss
P7	Yes	Yes	Moderate	T2	N0	Yes	Diffuse and strong	Moderate	LUL	Diffuse and strong
P8	Yes	Yes	Moderate	T3	N3	No	-	-	-	-
P9	Never	Yes	Moderate	T3	N2	No	Diffuse and strong	Poor	RUL	Diffuse and strong
P10	Yes	Yes	Moderate	T3	N3	Yes	Diffuse and strong	Moderate	LLL	Diffuse and strong
P11	Yes	Yes	Poor	T3	N2	Yes	Complete loss	Poor	LLL	Complete loss
P12	Never	Never	Moderate	T3	N1	No	Complete loss	Moderate	RML	Complete loss
P13	Yes	Yes	Well	T3	N2	No	Complete loss	Well	RUL	Complete loss
P14	Yes	Yes	Poor	T3	N1	No	Complete loss	Moderate	LUL	Diffuse and strong

ESCC, esophageal squamous cell carcinoma; LUSC, lung squamous cell carcinoma; LUL, left upper lobe; LLL, left lower lobe; RLL, right lower lobe; RML, right middle lobe; RUL, right upper lobe; LVI, lymphovascular invasion.
